# Electrophoretic deposition of magnesium oxide coating on micro-arc oxidized titanium for antibacterial activity and biocompatibility

**DOI:** 10.1186/s13018-023-04390-4

**Published:** 2023-11-27

**Authors:** Xinli Fan, Jiaheng Du, Yaohua Li, Ke Duan, Gangli Liu

**Affiliations:** 1 Department of Oral and Maxillofacial Surgery, School and Hospital of Stomatology, Shandong University & Shandong Key Laboratory of Oral Tissue Regeneration & Shandong Engineering Laboratory for Dental Materials and Oral Tissue Regeneration & Shandong Provincial Clinical Research Center for Oral Diseases, Cheeloo College of Medicine, No.44-1 Wenhua Road West, Jinan, 250012 Shandong China; 2 Sichuan Provincial Laboratory of Orthopaedic Engineering, Department of Bone and Joint Surgery, Affiliated Hospital of Southwest Medical University, 25 Taiping Rd, Luzhou, 646000 Sichuan China

**Keywords:** Dental implant, Titanium, Micro-arc oxidation, Electrophoretic deposition, Magnesium oxide, Antibacterial, *Porphyromonas gingivalis*

## Abstract

Titanium (Ti) dental implants face risks of early failure due to bacterial adhesion and biofilm formation. It is thus necessary to endow the implant surface with antibacterial ability. In this study, magnesium oxide (MgO) coatings were prepared on Ti by combining micro-arc oxidation (MAO) and electrophoretic deposition (EPD). The MgO nanoparticles homogeneously deposited on the microporous surface of MAO-treated Ti, yielding increasing coverage with the EPD time increased to 15 to 60 s. After co-culture with *Porphyromonas gingivalis* (*P. gingivalis*) for 24 h, 48 h, and 72 h, the coatings produced antibacterial rates of 4–53 %, 27–71 %, and 39–79 %, respectively, in a dose-dependent manner. Overall, EPD for 45 s offered satisfactory comprehensive performance, with an antibacterial rate 79 % at 72 h and a relative cell viability 85 % at 5 d. Electron and fluorescence microscopies revealed that, both the density of adherent bacterial adhesion on the surface and the proportion of viable bacteria decreased with the EPD time. The morphology of cells on the surface of each group was intact and there was no significant difference among the groups. These results show that, the MgO coating deposited on MAO-treated Ti by EPD had reasonably good in vitro antibacterial properties and cytocompatibility.

## Introduction

Titanium (Ti) dental implants are widely used to repair tooth loss. However, early infection remains a major risk of failure, with *Porphyromonas gingivalis* (*P. gingivalis*) being the leading pathogen [1–3]. Consequently, endowing dental implants with antibacterial capacities is clinically desirable.

Micro-arc oxidation (MAO) is a surface modification technique successfully applied to dental implants. In MAO, the implant is immersed in an electrolyte and a high voltage is applied, which creates local discharges (*i.e.*, arcs). The high temperature and pressure accompanying the arcs convert the implant surface into a titanium dioxide (TiO_2_) layer with roughness and porosity at micrometer dimension [4]. Biologically, this porous TiO_2_ layer provides satisfactory biocompatibility for adjacent bone regeneration and implant fixation [5]. Physically, this layer also offers a possible space for the storage and release of antimicrobial substances [6, 7]. For example, Jia et al. fabricated silver nanoparticles on MAO-modified Ti (MAO-Ti) surface, and achieved an antibacterial rate of 99.85 % against *Staphylococcus aureus* (*S. aureus*) [7]. However, silver is considerably cytotoxic to cells [8–10]. Therefore, it is still necessary to develop a implant surface that reduces bacterial infection without adversely affecting biocompatibility.

Magnesium (Mg) is an essential element for the human body (~ 20–30 g/adult), with a recommended daily intake of ~ 330 mg. Recently, Mg and several related compounds have been reported to possess antibacterial and ostegenic activities [11–13]. Lin et al. prepared MgO coating on Ti by magnetron sputtering; after co-culture with *P. gingivalis* for 24 h, the MgO coating produced antibacterial rates of 78.14–99.86 % [14]. Coelho et al. prepared MgO/hydroxyapatite composites and co-cultured them with three bacterial species for 24 h. They observed that, the adhesion and growth of the bacteria were all significantly inhibited [15].

Electrophoretic deposition (EPD) is a simple and efficient coating technique extensively investigated for the deposition of nano-sized particles on Ti implants to form osteogenic and antibacterial coatings [16]. Suntharavel et al. prepared nano-sized hydroxyapatite coating on Ti by EPD, and found the coating promoted the attachment and proliferation of osteoblasts after co-culture for 7 d [17]. Hickey et al. prepared nano-sized MgO coating on polylactic acid by EPD, and observed that the coating produced antibacterial rates of 64–90 % after co-culture with three bacterial species for 4 h [18].

Given the rough and porous nature of MAO-Ti surface and the technical advantages of EPD, it appears reasonable that, MAO-Ti may enable the entrapment-anchoring of nano-sized MgO deposit (i.e., coating) prepared by EPD. However, this has not been explored by available studies. The present study reported EPD of MgO on MAO-Ti and evaluated its in vitro antibacterial activity and biocompatibility.

## Materials and methods

### Micro-arc oxidation of titanium

Commercially pure Ti sheets (Grade 2, thickness 1 mm; Baoti Group, Baoji, Shanxi, China) were cut into 30 × 10 mm rectangular samples, abraded to 1200 grit with silicon carbide abrasive paper, etched in a mixture acid [3 % (w/w) hydrofluoric acid (Chuandong Chemical, Chongqing, China) and 5 % (w/w) nitric acid (Ghtech, Guangzhou, Guangzhou, China)] for 1 min, and sonicated in deionized water for 30 min. A cleaned Ti samples and a 316 stainless steel plate (100 × 20 mm) were installed on a fixture (Ti-stainless distance: 5 cm) and both partially immersed in an aqueous electrolyte [0.8 % (w/w) β-glycerophosphate sodium (Macklin, Shanghai, China), 5.9 % (w/w) calcium acetate (Macklin)] to serve as the positive and negative electrodes, respectively. A DC voltage of 300 V (STP-400 V/200A.D.R; Sanyang Instrument, Zhongshan, Guangdong, China) was applied between the electrodes for 30 s. The Ti sample was removed and sonicated repeatedly in deionized water.

### Electrophoretic deposition of nano-sized MgO on micro-arc oxidized titanium

Nano-sized MgO powder (0.5 g) (30 nm; Xinkang Advanced Materials, Changsha, Hunan, China) was suspended in 150 mL of acetone (Chuandong Chemical) and sonicated for 60 min at room temperature to form a suspension. An MAO-Ti sample (Sect. "Micro-arc oxidation of titanium") and a platinum plate (20 × 20 mm, Ledonlab, Shanghai, China) were partially immersed into the suspension to serve as the negative and positive electrodes, respectively. A DC voltage of 40 V was applied (Keithley, Shenzhen, Guangdong, China) for 0, 15, 30, 45, or 60 s [19]. The samples obtained were named EPD-0, EPD-15, EPD 30, EPD-45, and EPD-60, respectively.

### Physicochemical characterizations

Phase identification was performed by X-ray diffraction (XRD; CuKα, 40 kV, 20 mA; TD-3500, Tongda Instrument, Dandong, Liaoning, China). Surface morphology and elements present at the surface were studied by scanning electron microscopy (FE-SEM; JEOL JSM-7500F) and paired energy-dispersive spectroscopy (EDS, Bruker 1048).

### In vitro cytocompatibility

#### Cell isolation

Human gingival tissue samples were collected from a patient undergoing tooth extraction at Department of Oral and Maxillofacial Surgery, Stomatological Hospital of Shandong University. For experiments involving human tissue, informed consent has been obtained by patient Runzhe Yang. Primary human gingival fibroblasts (HGF) were isolated from the tissue samples by tissue block attachment [20] and routinely cultured (37 °C, 5 % CO_2_ −95 % air, 100 % relative humidity; Thermo Fisher 3111GP) in a standard medium [89 % high glucose Dulbecco’s modified eagle medium, 10 % fetal bovine serum (both Gibco), 1 % penicillin/streptomycin (Beyotime, Shanghai, China)]. This study was approved by Ethics Review Committee of Shandong University, and all experiments were performed in accordance with relevant guidelines and regulations.

#### Cytotoxicity assay

Samples were sterilized by dry heating (250 °C, 1 h) and placed in 24 well plates; 1 mL of the HGF suspension (1 × 10^5^ cells/mL) was pipetted on each sample and cultured for 1, 3, or 5 d. Then, 200 μL of CCK-8 reagent (Bioground, Chongqing, China) was added to each well and incubated for 2 h. Then, 100 μL of the liquid in each well was aspirated into a 96-well plate and measured for optical density (450 nm; Infinite M Nano microplate reader, Tecan, Männedorf, Switzerland). Additionally, 200 μL of CCK-8 reagent was added to wells containing 1 mL of HGF cell suspension but no sample and treated otherwise identically to serve as the control group. Finally, 200 μL of CCK-8 reagent was added to wells containing only 1 mL of culture medium (i.e., no cells nor sample) to serve as the blank group. The viability of cells was calculated by: Cell viability = [(OD_sample group_—OD_blank group_) / (OD_control group_ – OD_blank group_)] × 100%.

#### Live/dead staining

After above co-culture (Sect. "Cytotoxicity assay"), selected samples were rinsed with phosphate buffered saline (PBS), stained with Live/Dead cell imaging kits (Thermo Fisher R37601) for 15 min, and imaged under an inverted fluorescence microscope (Zeiss Axio Vert. A1). Unless otherwise specified, in following sections, all PBS rinsing procedures were 2 mL × 3.

#### Cell morphology

After culture for 5 d (Sect. "Cytotoxicity assay"), selected samples were rinsed with PBS, immersed in 4 % (v/v) paraformaldehyde (Biosharp, Hefei, Anhui, China) for 15 min, and rinsed with PBS. TritonX-100 [0.1 % (v/v); 500 μL; Beyotime] was added to each well, allowed to rest for 10 min to lyse the membrane, and rinsed with PBS. The cells were stained with phalloidin (Alexa Fluor 488, Thermo Fisher) and DAPI (4’, 6-diamidino-2-phenylin-dole) (Beyotime, China) following manufacturer instructions. Finally, the samples were observed under a laser scanning confocal microscope (Zeiss LSM980).

### In vitro antibacterial activity

#### Antibacterial assay

*P. gingivalis* (ATCC33277, HS1825, Jihebio, Shanghai, China) was made into single colonies. One colony was picked and cultured in 10 mL of Brain Heart Infusion Broth (HB8478) supplemented with vitamin K and hemin (all Hopebio, Qingdao, Shandong, China) for 24 h (37 °C, 150 rpm). The bacterial suspension was diluted to 1 × 10^5^ CFU/mL, inoculated uniformly onto sterilized samples preplaced in a 24-well plate, and incubated anaerobically for 24, 48, or 72 h. Subsequently, 2.5 mL of PBS was added to each well and blown with a pipette to dislodge bacteria from the sample; this process was repeated 4 times.

One millimeter of the liquid was collected from each well and combined with 9 mL of PBS. This combined liquid was diluted 10^5^-fold; 200 μL of the dilutent was inoculated on BHI agar (Hopebio) and incubated (37 °C) for 24 h; and the colonies formed were counted. The antibacterial rate was calculated by: antibacterial rate = [(control group- sample group)/control group] × 100%.

In a separate experiment, bactericidal efficacies of the samples were evaluated by measuring total superoxide dismutase (SOD) activities as follows. The bacteria were inoculated on the samples and cultured identically. The culture medium was blown, and 60 μL was aspirated to an centrifugation tube and assayed for with a commercial kit (Beyotime, S0109) following manufactuer’s instructions.

#### Bacterial morphology

After culture for 72 h, the samples were rinsed with PBS to eliminate non-adherent bacteria, fixed with 4 % (v/v) paraformaldehyde for 24 h, dehydrated by immersion in ethanol series (50, 70, 80, 90 % once, 100 % twice, 15 min each; all v/v), and studied by SEM.

#### Live/dead staining

Selected above co-cultured samples (Sect. "Antibacterial assay") were rinsed with PBS, stained with LIVE/DEAD BacLight Bacterial Viability Kit (Thermo Fisher L7012) following manufacturer instructions, and observed under the inverted fluorescence microscope.

### Statistical analysis

Data were analyzed by one-way analysis of variance (ANOVA, SPSS 16.0, SPSS, Chicago, IL, USA) and Tukey multiple comparison test. A *p* < 0.05 was considered statistically significant.

## Result

### Morphology, surface elements, and phase

**Fig. 1:**
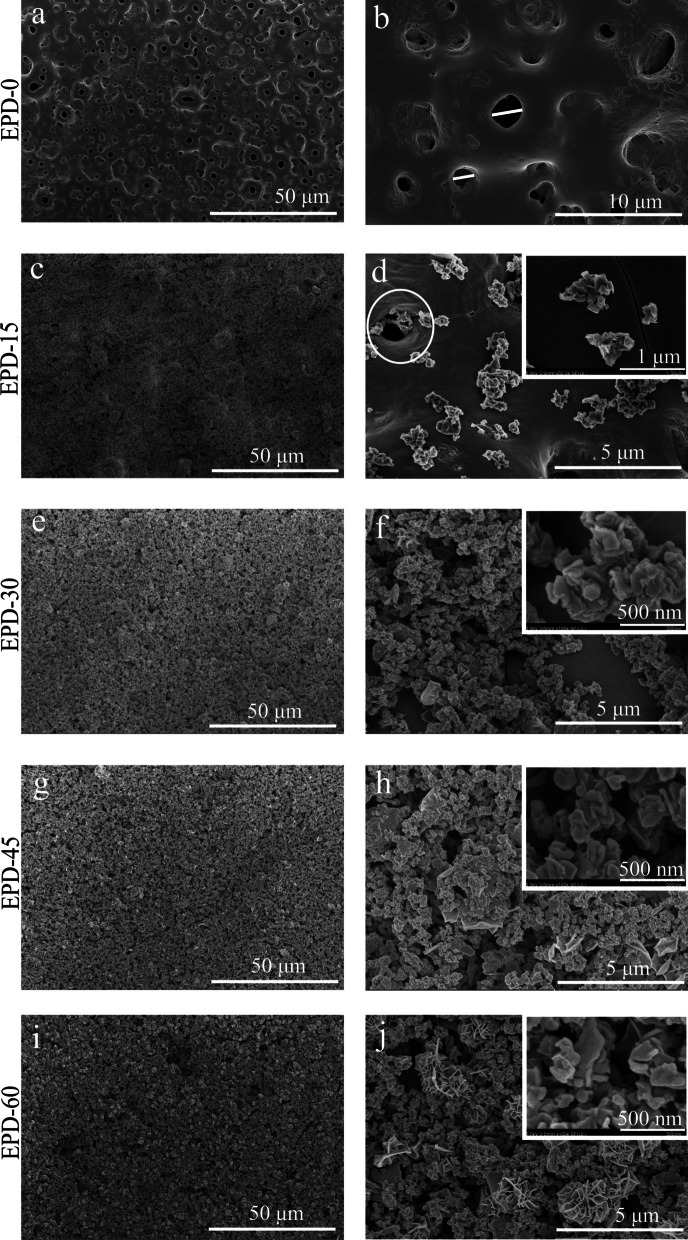
**a–j** SEM images of each samples. **a**, **b** EPD-0; **c**, **d** EPD-15; **e**, **f** EPD-30; **g**, **h** EPD-45; **i**, **j** EPD-60

**Fig. 2:**
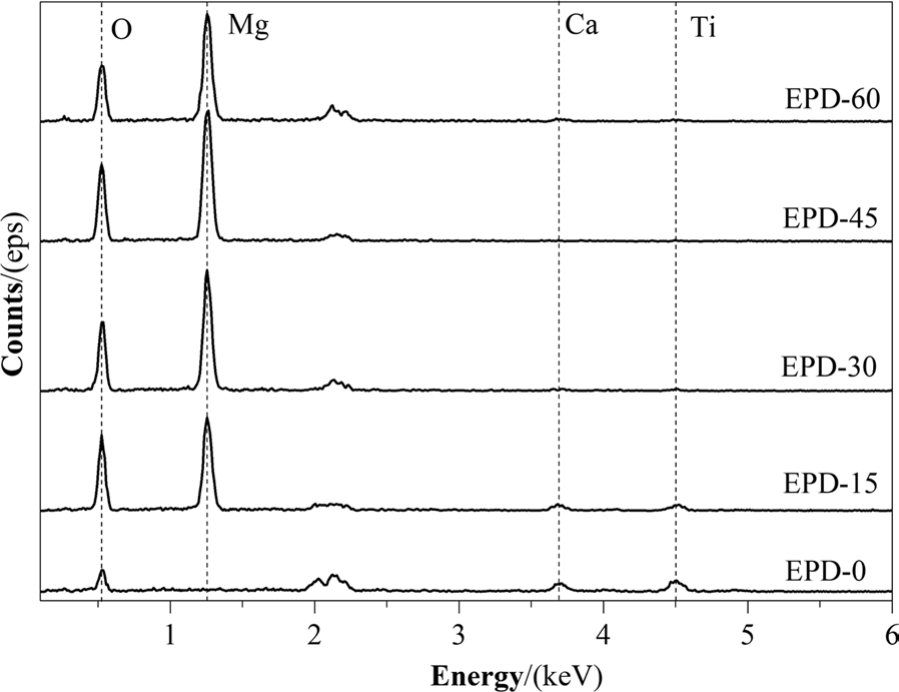
EDS spectra of each samples

**Fig. 3:**
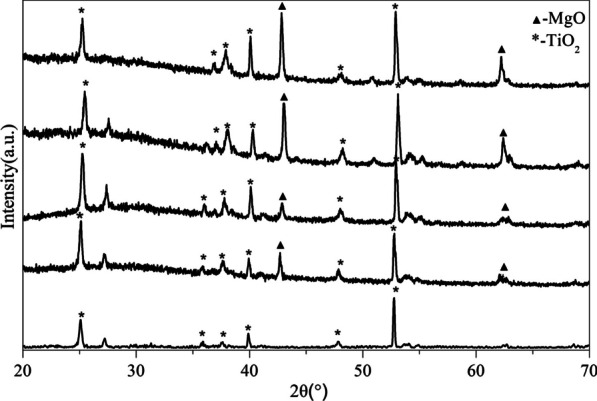
XRD spectra of each samples

SEM observation revealed a large number of crater-like micropores on the surface of EPD-0 (i.e., micro-arc oxidized Ti) (Fig. 1a, b), with a diameter of approximately 2–6 μm. After 15 – 60 s of EPD (Fig. 1c–j), aggregated nano-sized MgO particles were deposited its surface. Surface coverage of the aggregates increased with EPD time, with EPD-45 and EPD-60 reaching nearly completely coverage (Fig. 1g–j). On EPD-15, some aggregates were located inside micropores (Fig. 1c, d). EDS found Oxygen (O), Calcium (Ca), Ti on the surface of EPD-0 (Fig. 2). In comparison, Mg was additionally detected from EPD-15 to EPD-60, yielding increasing peak intensities with EPD time relative to the peaks of O, Ca, and Ti. XRD found TiO_2_ from all samples (Fig. 3). MgO was detected from EPD-15 to EPD-60, with increasing peak intensities with EPD time relative to those of TiO_2_.

### Cytocompatibility

**Fig. 4:**
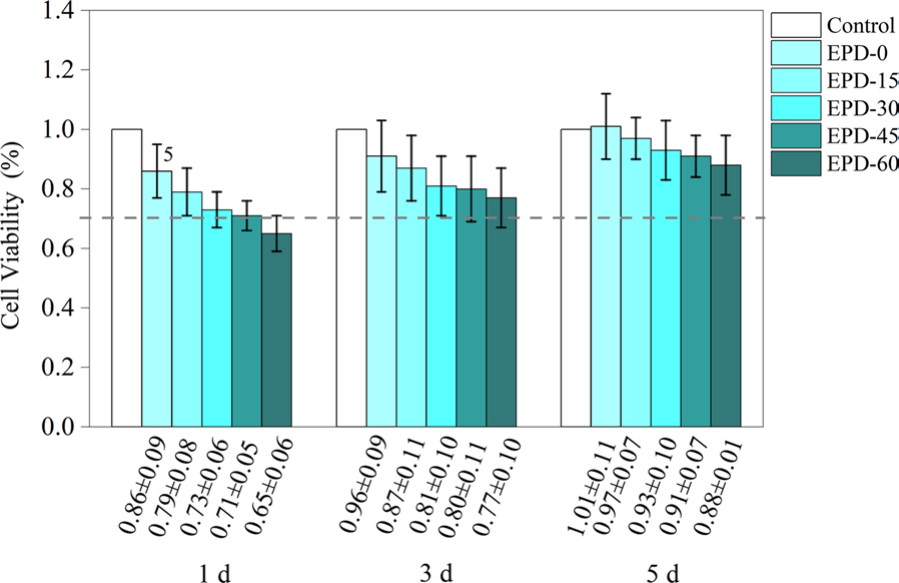
Viability of HGFs after co-culture on samples for 1, 3 and 5 d. Numbers 5 indicates a statistically significant difference vs. EPD-60 (*p* < 0.05)

Figure 4 depicts the relative survival rates of HGF cells cultured on various surfaces. Between 1 and 5 d, the relative survival rate of each group increased with the time of culture. At each time point, it decreased with the EPD time. On day 1, the survival rates of groups EPD-15 to EPD-60 were reduced by 8 %, 15 %, 17 %, and 24 %, respectively, compared with EPD-0. With the progression of culture time, the difference between groups narrowed. On day 5, they were reduced by 4 %, 8 %, 10 %, and 13 % (vs. EPD-0), respectively. On day 1, the difference between EPD-0 and EPD-60 was statistically significant (*p* = 0.004). No statistically significant difference was detected at other time points or between other group pairs. According to ISO 10993–5[21], a relative survival rate ≥ 70% is considered non-cytotoxic. Based on this criterion, EPD-60 was cytotoxic (survival rate = 65%) only on day 1. No other group was found cytotoxic at any time point.

**Fig. 5:**
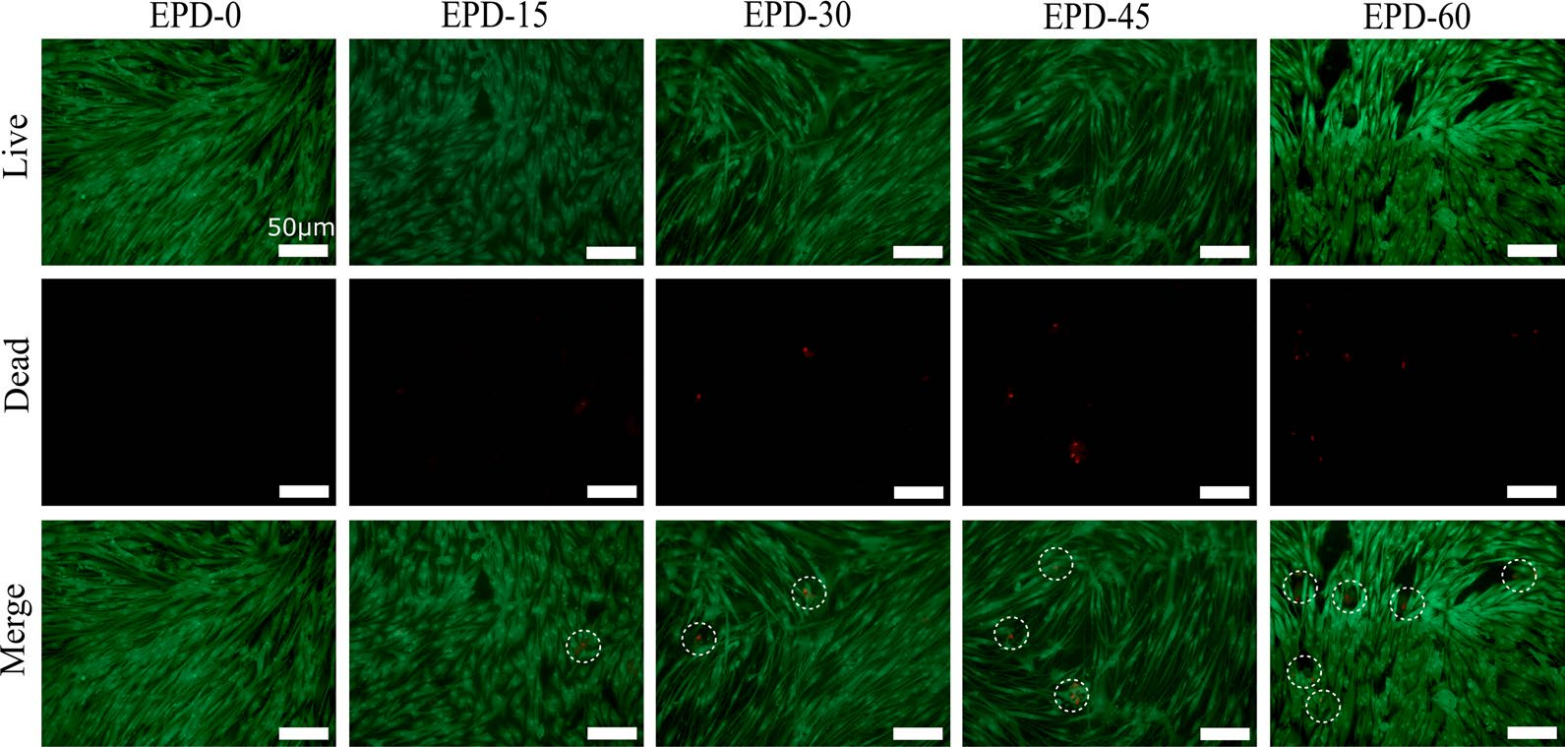
Fluorescent micrographs of Live/Dead-stained HGFs seeded on samples after co-culture for 5 d. Red pixels: dead cells; green pixels: viable cells. All scale bars: 50 μm. White circles: dead cells

**Fig. 6:**
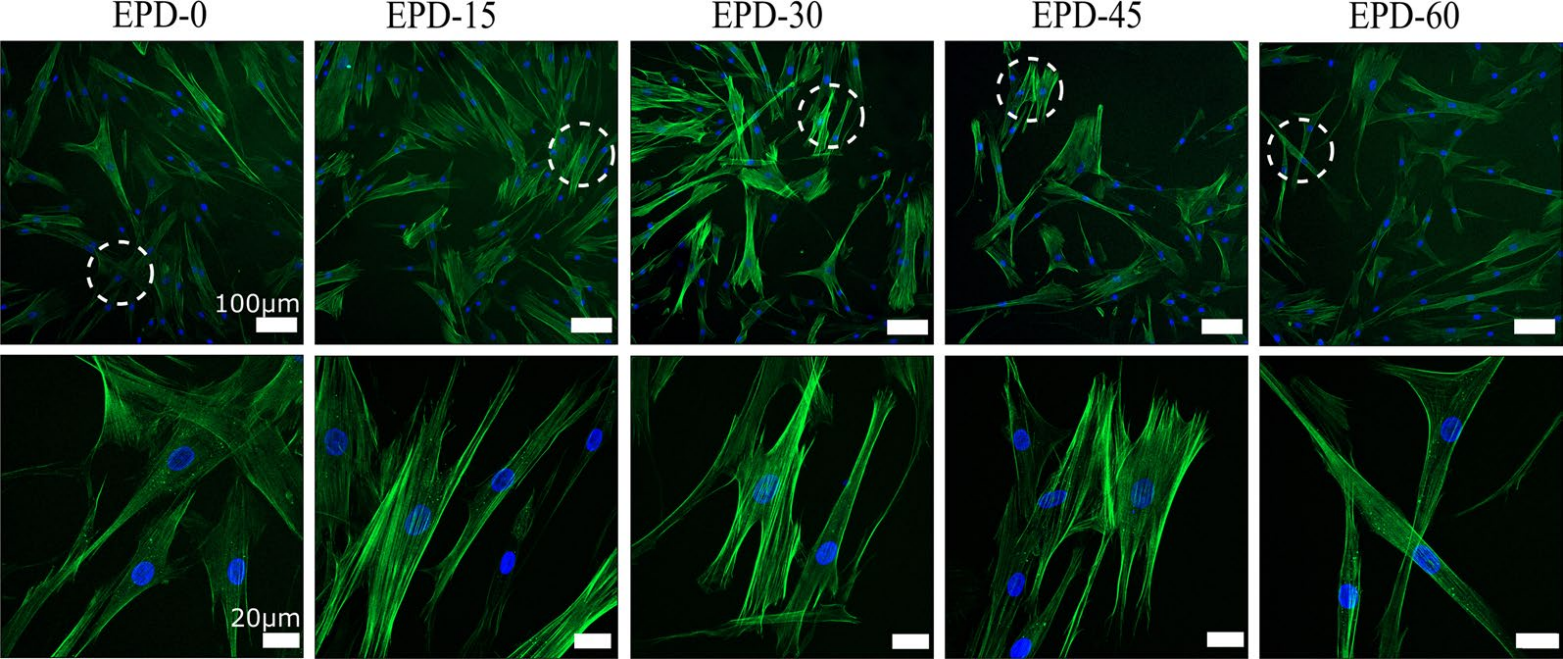
Low and high-power fluorescence micrographs of HGFs seeded on samples after co-culture for 5 d. HGFs stained for cykoskeleton fibers (green pixels) and nuclei (blue pixels). All low-power scale bars: 20 μm; all high-power scale bar: 100 μm. White circles: regions shown at a higher power view in lower panel

Live/Dead staining of samples cultured for 5 d (Fig. 5) revealed a small number of dead cells (red pixels) on EPD-15 to EPD-60, with increasing numbers with the EPD time. After culture for 5 d, the capacity of HGF cells to develop cytoskeleton was examined by staining of cytoskeletal actinfibers (green fluorescence) and nuclei (bule fluorescence) (Fig. 6). It was observed that, each group formed fibrous cytoskeletons distributing throughout the cytoplasm, indicating that the MgO coatings did not disturb the organization of cytoskeleton.

### Antibacterial activity

**Fig. 7:**
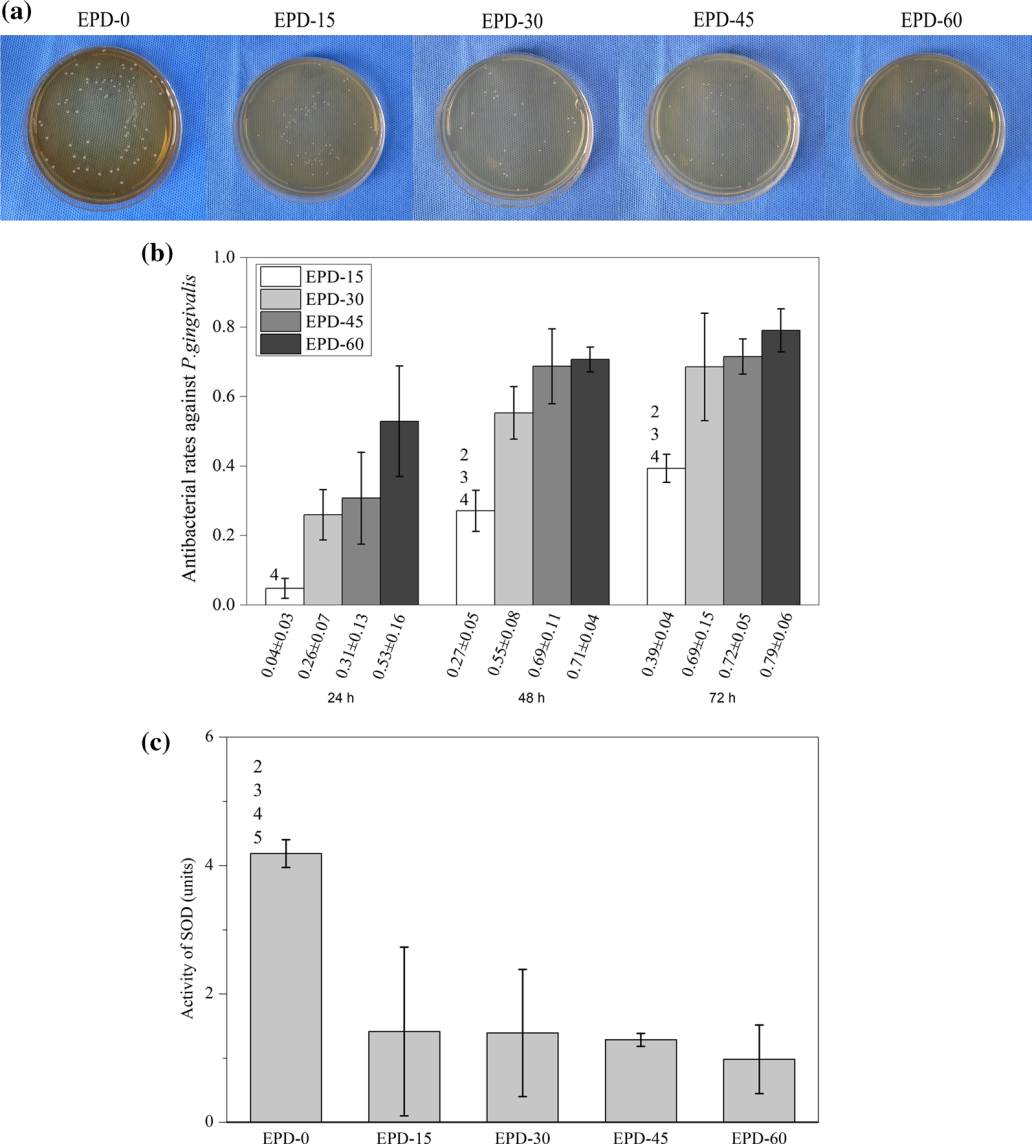
**a** Representative photographs of colonies formed on samples after co-culture with *P. gingivalis* for 24 h; **b** bacterial colony numbers, numbers 2, 3, 4 indicate statistically significant differences (*p* < 0.05) vs. EPD-30, EPD-45, and EPD-60, respectively; **c** SOD activity value. Numbers 2, 3, 4, 5 indicate statistically significant differences (*p* < 0.05) vs. EPD-15, EPD-30, EPD-45, and EPD-60, respectively

Figure 7a shows representative photographs of the bacterial colonies formed on agar plates 1 d after inoculation of diluted *P. gingivalis* suspensions derived from each group after co-culture for 72 h. It was evident that, the number of colonies decreased sequentially from EPD-0 to EPD-60. Calculations showed that, the antibacterial rates of EPD-15 to EPD-60 (Fig. 7b) were 4 ± 3 %, 26 ± 7 %, 31 ± 13 %, and 53 ± 16 % at 24 h, 27 ± 5 %, 55 ± 8 %, 69 ± 11 %, and 71 ± 4 % at 48 h, and 39 ± 4 %, 69 ± 15 %, 72 ± 5 %, and 79 ± 6 % at 72 h, respectively. At 24 h, the difference between EPD-15 and EPD-60 was statistically significant difference (*p* = 0.003). At 48 and 72 h, ANOVA found statistically significant differences between EPD-15 and EPD-30 to EPD-60 (*p* < 0.05).

At 24 h (Fig. 7c), the SOD activity measured from the 5 groups were 4.2 ± 0.2, 1.4 ± 1.3, 1.4 ± 1.0, 1.3 ± 0.1, and 1.0 ± 0.5, units, respectively. The differences between EPD-0 and the other four groups were statistically significant (all *p* < 0.05).

**Fig. 8:**
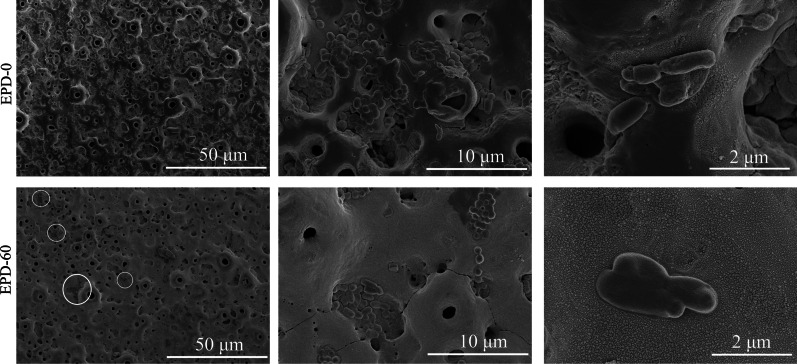
SEM micrographs of EPD-0 and EPD-60 after co-culture with *P. gingivalis* for 72 h. White circles: *P. gingivalis* cells

**Fig. 9:**
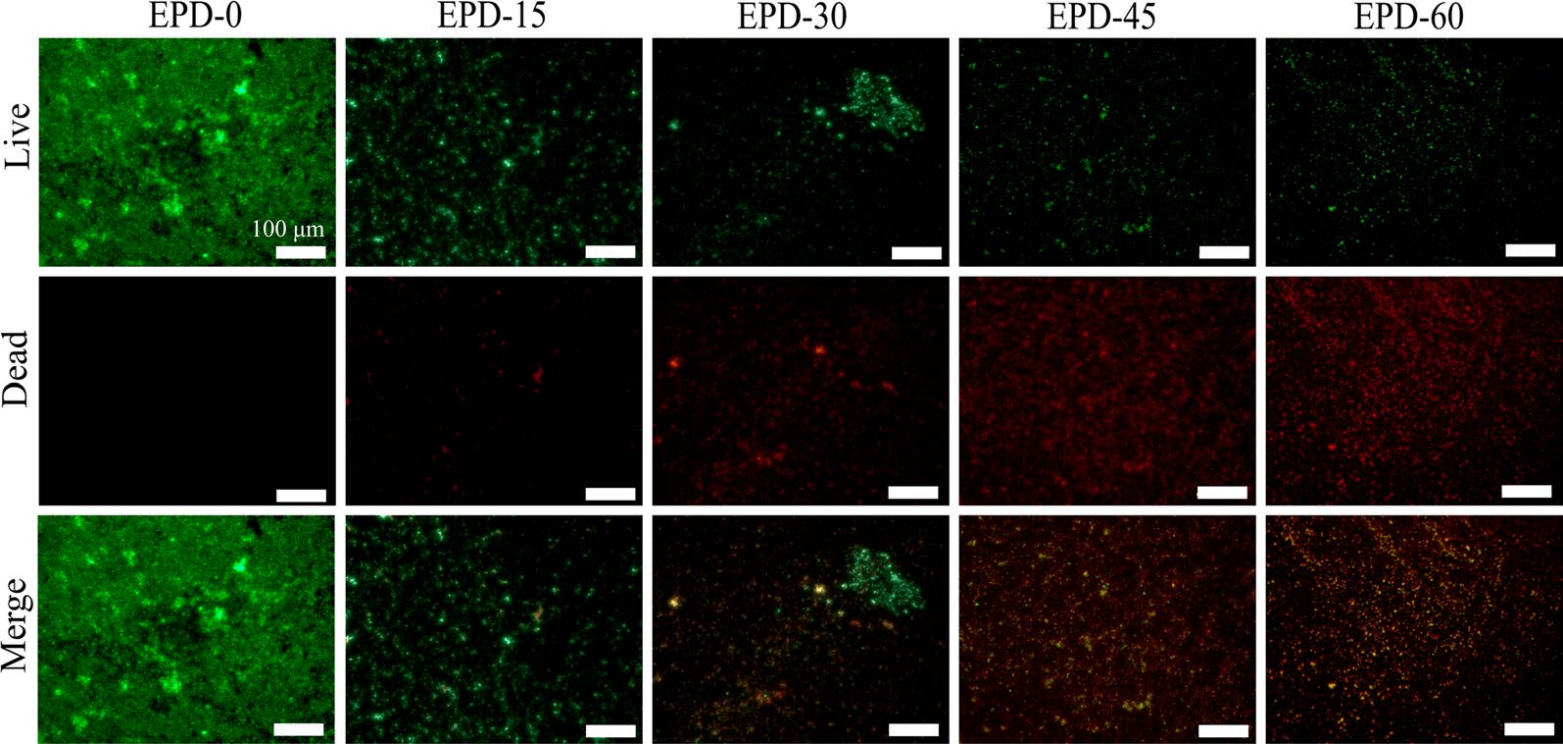
Micrographs of samples after Live/Dead fluorescent staining on co-cultured with *P. gingivalis* for 10 h. Red pixels: dead *P.* *gingivalis* cells; green pixels: viable *P. gingivalis* cells. All scale bars: 100 μm

SEM observations of samples co-cultured for 72 h found that (Fig. 8), a large number of rod-like bacteria adhered to the surface of EPD-0, many located in the micropores, covering ~ 15 of the surface. In comparison, substantially fewer bacteria adhered to EPD-60, covering ~ 3 %. Live/dead staining of samples co-cultured for 72 h (Fig. 9) detected practically no dead bacterial cells (would be red pixels) on EPD-0. In contrast, with the increase of EPD time, the other 4 groups gave increasingly intense fluorescence of dead bacterial cells and generally weakening fluorescence of viable ones.

## Discussion

In this study, we prepared nano-sized MgO coatings on Ti by combining MAO with EPD for dental implantation. After MAO treatment of Ti, MgO was deposited on it from a MgO-acetone suspension. Acetone has advantages of low viscosity and high dielectric coefficient [22], allowing easy deposition of MgO nanoparticles and satisfactory coating uniformity. Compared with other coating methods for MgO (e.g., sol–gel, chemical or physical vapor deposition), EPD offers unique advantages of simple equipment, rapid preparation, and uniform coating, and is widely used for coating preparation [17, 23, 24].

All MgO-coated samples showed antibacterial activities against *P. gingivalis* with increasing antibacterial rates with EPD time, with EPD-60 attaining 79 % at 72 h (Fig. 7b). The reduction in SOD levels in all EPD-treated groups also corroborates the killing of the bacteria [25]. The reduction did not quantitatively correlate with the results of broth dilution. This may be partly related to experimental methodologies and errors, and further studies are required to better understand the roles of SOD and other antioxidases/enzymes in MgO-triggered bacterial destruction.

Although the antibacterial rates observed in the present study are lower than those reported for silver nanoparticles and antibiotics, the MgO coatings offer advantages of low toxicity and inducing no drug resistance. In addition, the EPD technique is applicable to a variety of coating materials. Therefore, in future studies, we expect to use EPD to deposit composite coatings of MgO with other materials on Ti implants to further enhance their short-term antibacterial activity and bioactivity. Sreekanth et al. combined EPD with plasma electrolytic oxidation to fabricate a composite coating of MgO/hydroxyapatite on magnesium alloy, and found that the coating significantly improved the bioactivity of the Mg alloy [26]. Many studies have reported antibacterial properties of MgO. Makhluf et al. synthesized MgO nanoparticles (8–23 nm) by microwave-assisted reactions, and found 99 % and 95 % antibacterial activities against both *Escherichia coli* (*E. coli*) and *S. aureus*, with increasing rates recorded from smaller particles [27]. However, the antibacterial mechanisms of MgO remain inconclusive. Some studies [28–30] suggested it to be related to reactive oxygen species (ROS) damage and increase in pH. It was reported that, MgO undergoes catalytic reactions with O_2_ to superoxide anions (O_2_^−^) [28]. MgO also reacts with water to generate Mg(OH)_2_, which creates an alkaline environment to likely cause bacterial membrane damage and death [29]. Dong et al. co-cultured Mg(OH)_2_ with *E. coli*, and observed that OH^−^ and Mg^2+^ ions in Mg(OH)_2_ water suspension were found not to be the reason for killing [30]. Huang et al*.* fabricated MgO particles of various size ranges, co-cultured them with two bacterial species, and found increased antibacterial effects with decreasing particle sizes. They suggested that, small particles (with larger surface areas) generated higher concentrations of O_2_^−^, which disrupted the bacterial cell membrane [31]. The mechanism of the coating in this study is still under investigation, and the results will be reported in the future.

Upon co-culturing the samples with HGF cells, a moderate cytotoxicity was observed on day 1 (Fig. 4); this may be related to factors such as the increase in pH value and ROS production. Nevertheless, the cytotoxicity decreased with the incubation time; this may be related to the stable incubation environment and the decomposition of ROS. Consistent with our findings, many other studies have reported the biocompatibility of MgO. Li et al. fabricated polymethylmethacrylate containing MgO and cultured extracts with MC3T3-E1 for 1–7 d; they found that the viability of cells exposed to the extract derived from the MgO-containing cement was significantly higher than those exposed to that from the MgO-free one [32]. Yu et al. prepared MgO coating on Ti by a sol–gel method and co-cultured it with osteoblasts for 5 d; they found that the MgO coating yielded improved biocompatibility and alkaline phosphatase activity than did the uncoated Ti [33]. The in vivo antibacterial and osteogenic properties of the coating in animal models have not been evaluated in this study, and further studies are needed to address these limitations and further explore the properties of the coating.

## Conclusion

The combination of MAO and EPD provides a simple, rapid, and readily adaptable approach to fabricate MgO coatings on Ti dental implants. The resultant coatings showed good in vitro antibacterial property and biocompatibility. The coating prepared by EPD for 45 s showed great comprehensive performance, giving a antibacterial rate 79 % at 72 h and relative cell viability 85 % at 5 d. Future studies will investigate the antibacterial properties, antibacterial mechanism and osteogenic properties of the coating.

## Data Availability

The datasets generated during and/or analysed during the current study are available from the corresponding author on reasonable request.
